# Stem Cell Transplant in Immune-deficiency–associated Vaccine-derived Poliovirus

**DOI:** 10.1093/ofid/ofad678

**Published:** 2024-01-17

**Authors:** Heena Ranchod, Wayne Howard, Adele Roux, Walda van Zyl, Pieter Ekermans, Sylvia van den Berg, Lerato Seakamela, Koketso Makua, Mukhlid Yousif, Rosinah Sibiya, Heleen Du Plessis, Emmanuel Phalane, Kerrigan McCarthy, Shelina Moonsamy, David Reynders, Jeffrey Hincks, Melinda S Suchard, Nicolette M du Plessis

**Affiliations:** Centre for Vaccines and Immunology, National Institute for Communicable Diseases, Johannesburg, South Africa; Department of Chemical Pathology, School of Pathology, Faculty of Health Sciences, University of the Witwatersrand, Johannesburg, South Africa; Centre for Vaccines and Immunology, National Institute for Communicable Diseases, Johannesburg, South Africa; Department of Chemical Pathology, School of Pathology, Faculty of Health Sciences, University of the Witwatersrand, Johannesburg, South Africa; Dr Adéle Roux Practice, Life Groenkloof Hospital, Pretoria, South Africa; Department of Medical Virology, Faculty of Health Sciences, University of Pretoria, National Health Laboratory Services—Tshwane Academic Division, Pretoria, South Africa; Department of Medical Microbiology, Ampath Laboratories, Pretoria, South Africa; Department of Immunology, Ampath Laboratories, Pretoria, South Africa; Centre for Vaccines and Immunology, National Institute for Communicable Diseases, Johannesburg, South Africa; Centre for Vaccines and Immunology, National Institute for Communicable Diseases, Johannesburg, South Africa; Centre for Vaccines and Immunology, National Institute for Communicable Diseases, Johannesburg, South Africa; Department of Virology, School of Pathology, Faculty of Health Sciences, University of the Witwatersrand, Johannesburg, South Africa; Centre for Vaccines and Immunology, National Institute for Communicable Diseases, Johannesburg, South Africa; Centre for Vaccines and Immunology, National Institute for Communicable Diseases, Johannesburg, South Africa; Centre for Vaccines and Immunology, National Institute for Communicable Diseases, Johannesburg, South Africa; Department of Virology, School of Pathology, Faculty of Health Sciences, University of the Witwatersrand, Johannesburg, South Africa; Centre for Vaccines and Immunology, National Institute for Communicable Diseases, Johannesburg, South Africa; Centre for Vaccines and Immunology, National Institute for Communicable Diseases, Johannesburg, South Africa; Department of Paediatrics, Faculty of Health Sciences, University of Pretoria, Pretoria, South Africa; ViroDefense, Chevy Chase, Maryland, USA; Centre for Vaccines and Immunology, National Institute for Communicable Diseases, Johannesburg, South Africa; Department of Chemical Pathology, School of Pathology, Faculty of Health Sciences, University of the Witwatersrand, Johannesburg, South Africa; Department of Paediatrics, Faculty of Health Sciences, University of Pretoria, Pretoria, South Africa

**Keywords:** acute flaccid paralysis, disseminated BCG, immunodeficiency, intravenous immune globulin, IRIS, iVDPV, pocapavir, polio, poliomyelitis, RAG, RAG, SCID, stem cell transplant, transplant, vaccine-associated, vaccine-derived poliovirus, VAPP

## Abstract

Patients with severe primary immunodeficiency are at risk for complications from live-attenuated vaccines. Here, we report a case of a vaccine-associated paralytic polio and Bacille Calmette-Guérin disease in a 6-month-old girl with severe combined immunodeficiency resulting from homozygous recombinant activating gene 1 deficiency. The patient was successfully treated with intravenous immunoglobulins and oral pocapavir for poliovirus, and antimycobacterial therapy for regional Bacille Calmette-Guérin disease, allowing stem cell transplant. Following transplantation, poliovirus type 3 with 13 mutations was detected from cerebrospinal fluid but not from stool, indicating ongoing viral evolution in the central nervous system despite pocapavir treatment. Clinical improvement and immune reconstitution allowed the patient to be successfully discharged with no further detection of poliovirus.

## CASE IN DISCUSSION

The patient, a 6-month-old female born in March 2021, was asymptomatic for the first 4 months of life. The child had no family history nor early indication of an underlying immunodeficiency. Per the South African Expanded Programme on Immunisation, she received Bacillus Calmette-Guérin (BCG) at birth and oral polio vaccine (OPV) at birth and 6 weeks of age as well as other age-appropriate vaccinations until 5 months of age when she was hospitalized with rhinovirus-associated bronchiolitis. Two weeks later, she had persistent respiratory symptoms with a worsening nocturnal cough and chest x-ray imaging suggestive of interstitial pneumonitis. During readmission to the hospital, nasopharyngeal aspirate was positive for rhinovirus and enterovirus by polymerase chain reaction (PCR). Because of the interstitial lung pattern and ongoing hypoxia, *Pneumocystis jirovecii* (PCP) was suspected, with the (1→3)-β-D-Glucan level greater than 280 pg/mL, but nasopharyngeal aspirate was negative for PCP by PCR. Tests for pulmonary *Mycobacterium tuberculosis* complex disease were negative (negative Mantoux test, negative sputum with tuberculosis PCR on the BD Max platform, and negative sputum *Mycobacterium tuberculosis* complex culture). The child was treated with high-flow oxygen but remained in respiratory distress with a respiratory rate of around 100 breaths/min despite oxygen saturation of 100%. The patient's antibiotics included ceftriaxone, which was escalated to ertapenem for suspected nosocomial pneumonia, and oral steroids. Cotrimoxazole and micafungin were added for suspected PCP and nosocomial fungal infection. Antibiotics were changed to meropenem and linezolid after the patient's condition worsened.

On 11 September 2021, the child's father noted decreased use of her left hand. Acute flaccid paralysis was clinically apparent, with decreased tone, poor power of 1 of 5, and absent reflexes upon examination of the left arm. Magnetic resonance imaging and Doppler scans of her brain and left arm showed no space-occupying lesions nor vascular abnormalities. Lumbar puncture revealed normal protein and glucose value with 10 mononuclear cells/µL, 1 erythrocyte/µL, and no polymorphonuclear cells. No viral or bacterial pathogens were isolated. The National Institute of Communicable Diseases was therefore notified of case of acute flaccid paralysis. Stool samples were sent for polio isolation on 15 September 2021, with multiple follow-up samples (Table 1).

**Table 1.** Polio Virus Results From Patient Samples

Sample Collection Date	Time Interval Between Initial Sample and Collection of New Sample	Sample Type	Cell Culture Result	ITD Result	Sequencing Result	No. of Mutations in the VP1 Region	% Similarity to Sabin 3
15/09/2021	Day 1	Stool	Suspected poliovirus	PV3 SL	P3 Sabin	4	99.56
15/09/2021	Day 1	CSF	ND	ND	Negative	ND	ND
16/09/2021	Day 2	Stool	Suspected poliovirus	PV3 SL	P3 Sabin	4	99.56
26/09/2021	Day 11	Stool	Suspected poliovirus	PV3 SL	P3 Sabin	7	99.22
29/10/2021	Day 14	Stool	Suspected poliovirus	PV3 SL	P3 Sabin	5	99.44
07/10/2021	Day 22	Stool	Suspected poliovirus	PV3 SL	P3 Sabin	6	99.33
14/10/2021	Day 29	Stool	Suspected poliovirus	PV3 SL	P3 Sabin	8	99.11
21/10/2021	Day 37	Stool	Negative	Zero	Zero	Zero	Zero
27/10/2021	Day 43	Stool	Negative	Zero	Zero	Zero	Zero
28/10/2021	Day 44	Stool	Negative	Zero	Zero	Zero	Zero
01/11/2021	Day 48^a^	Stool	Negative	Zero	Zero	Zero	Zero
02/11/2021	Day 49	Stool	Negative	Zero	Zero	Zero	Zero
03/11/2021	Day 50	Stool	Negative	Zero	Zero	Zero	Zero
08/11/2021	Day 55	Stool	Negative	Zero	Zero	Zero	Zero
08/11/2021	Day 55	CSF	ND	ND	Negative	ND	ND
09/11/2021	Day 56	Stool	Negative	Zero	Zero	Zero	Zero
11/11/2021	Day 58	Stool	Negative	Zero	Zero	Zero	Zero
12/11/2021	Day 59	Stool	Negative	Zero	Zero	Zero	Zero
13/11/2021	Day 60	Stool	Negative	Zero	Zero	Zero	Zero
28/11/2021	Day 75	Stool	Negative	Zero	Zero	Zero	Zero
07/12/2021	Day 84	Stool	Negative	Zero	Zero	Zero	Zero
28/02/2022	Day 168—patient underwent bone marrow transplant						
29/03/2022	Day 198A	CSF	Negative	ND	P3 Sabin	13	98.56
30/03/2022	Day 198B	CSF	Negative	ND	P3 Sabin	11	98.78

Day 1 was considered the date of collection of the first stool specimen.

Abbreviations: CSF, cerebrospinal fluid; ITD, intratypic differentiation; ND, not done; P3, poliovirus serotype 3; PV3 SL, poliovirus 3 Sabin-like; VP1, viral protein region 1.

^a^Pocapavir start date was on day 48 and ended on day 61.

Further immunological investigations were conducted because of persistent lymphopenia and the progressive nature of her clinical picture. Markedly decreased levels of immunoglobulins A, G, and M, a total lymphocyte count of 300 cells/µL, and severely decreased T- and B-lymphocyte subsets were noted, and a diagnosis of T-B-NK+ severe combined immunodeficiency (SCID) was made (Table 2). The child tested negative for HIV.SCID was later confirmed by the identification of 2 pathogenic variants in RAG1, c.1677G > T (p.Arg559Ser) (Invitae Diagnostics, San Fransisco, California).

**Table 2.** Investigation for Primary Immune Deficiency

Cell Type	Absolute Count	Reference Range
Total lymphocyte count	**300 cells/µL**	1800–18 700 cells/µL
Immunoglobulin G	**0.55 g/L**	3.46–6.58 g/L
Immunoglobulin A	**<0.07 g/L**	0.13–0.75 g/L
Immunoglobulin M	**0.08 g/L**	0.03–0.75 g/L
Total T cells (CD3+)	**21 cells/µL**	1900–5900 cells/µL
Helper T cells (CD4+)	**19 cells/µL**	1400–4300 cells/µL
Cytotoxic T cells (CD8+)	**11 cells/µL**	500–1700 cells/µL
Natural killer cells (CD16+cD56+)	272 cells/µL	160–950 cells/µL
B cells (CD19+)	**1 cell/µL**	610–2600 cells/µL
Recent thymic emigrants	**Absent**	…
Kappa deleting recombination excision circles	**Absent**	…

Bold font indicates abnormal values.

A Sabin-like poliovirus serotype 3 was isolated from stool. Initial Sanger sequencing revealed 4 mutations in the VP1 region, in keeping with a vaccine strain. Isolation precautions (contact and airborne) were initiated, as were daily intravenous immune globulin replacement and breastmilk feeds to provide maternal antibodies. Her parents were vaccinated with quadrivalent vaccine (tetanus, diphtheria, acellular pertussis, inactivated polio) and SARS-CoV-2 vaccines to prevent potential transmission to the child.

The child's condition worsened over time, requiring admission to the intensive care unit, where she was intubated and ventilated. Following administration of intravenous immunoglobulin, she developed osmotic nephrosis and vomiting as adverse effects and required antidiuretic therapy and total parenteral nutrition as support.

Following intensive care unit admission, the child's BCG scar appeared red and enlarged with a right axillary lymphadenopathy. Although mycobacterial investigations could not prove regional BCG disease, the patient was empirically treated with antimycobacterial therapy, namely rifampicin, isoniazid, pyrazinamide, ethambutol, and levofloxacin Workup for bone marrow transplant was initiated.

For just over 1 month, poliovirus was detected in stool with mutations accumulating in the VP1 region (Table 1, still numbering fewer than 10 mutations). Pocapavir, an investigational drug used for the treatment of enteroviruses, was procured through a Section 21 application for unlicensed products (ViroDefense, Chevy Chase, Maryland). Stool tested negative for poliovirus following a bout of diarrhea; however, pocapavir administration was commenced at the recommended dose and given for 2 weeks.

Stool samples during this time continued to test negative for poliovirus (Table 1). She received an allogeneic stem cell transplant in February 2022 at the age of 11 months.

Approximately 1 month after the successful bone marrow transplant, the patient developed new symptoms of whole-body fasciculations. Repeat lumbar puncture results revealed low positive PCR results for enterovirus on the cerebrospinal fluid (CSF) samples using the fast-track diagnostics real-time PCR kit for viral meningitis (Labgene Scientific) on the 480 light cycler (Roche). Results of the CSF samples were confirmed by sequencing at a second laboratory, the National Institute of Communicable Diseases [1–3]. Other tests on CSF were negative including cytomegalovirus, human herpesvirus-6, John Cunningham virus, viral meningitis, *Listeria monocytogenes*, *Mycobacterium tuberculosis*, and fungal culture.

**Figure 1.**
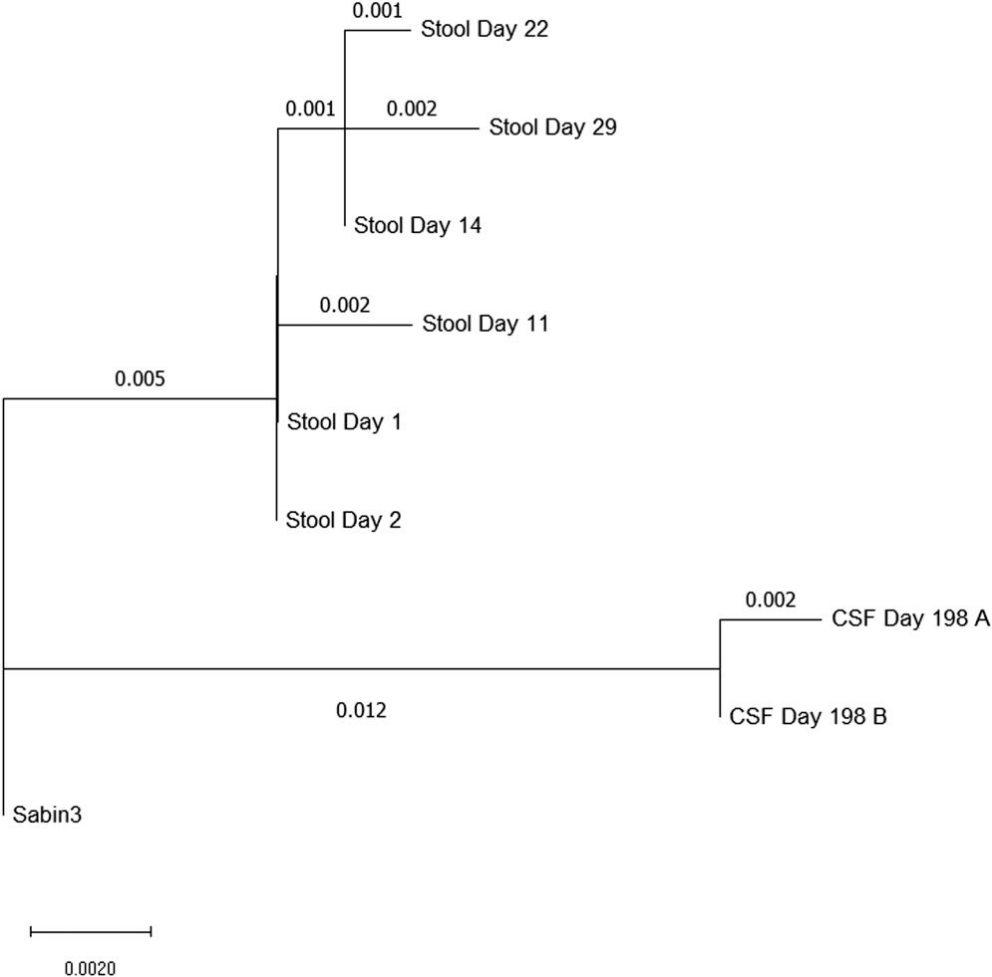
Neighbor-Joining tree illustrating the accumulation of poliovirus mutations in the patient over time. The evolutionary history was inferred using the Neighbor-Joining method [4] with the tree rooted to Sabin 3. The percentage of replicate trees in which the associated taxa clustered together in the bootstrap test (1000 replicates) are shown next to the branches [5]. The tree is drawn to scale, with branch lengths in the same units as those of the evolutionary distances used to infer the phylogenetic tree. The evolutionary distances were computed using the maximum composite likelihood method [6] and are in the units of the number of base substitutions per site. This analysis involved 9 nucleotide sequences. Codon positions included were 1st+2nd+3rd+ noncoding. All ambiguous positions were removed for each sequence pair (pairwise deletion option). There was a total of 900 positions in the final dataset. Evolutionary analyses were conducted in MEGA11.

Genotyping results from the CSF sample amplicons showed Sabin-like poliovirus serotype 3 with 11 and 13 mutations in the VP1 region, respectively, classified as a vaccine-derived poliovirus. The evolution of the virus is shown in Figure 1.

Additional analyses of the genotyping data found the following amino acid substitutions (L, leucine; I, isoleucine; A, alanine; T, threonine; N, asparagine; D, aspartic acid; K, lysine; R, arginine; V, valine; S, serine; E, glutamic acid)—CSF: L18I, A54T, N142D, K166R, T232A, V256I; and stools: D23N, A54V, R286S, E294K. No common amino acids were found between the CSF and stool samples; however, position 54 was T in CSF and V in stools. Furthermore, none of the substitutions has been previously reported as associated with neurovirulence [7, 8].

The patient was discharged and followed up as an outpatient on monthly intravenous immune globulin. She was last seen at age 2 years with poor weight gain and neurodevelopmental delay. Her left arm shows residual paresis with minimal function. She has also required repeat treatment for BCG manifestations as part of further immune reconstitution.

## DISCUSSION

Poliomyelitis (polio) is a disease caused by the poliovirus, a member of the Enterovirus C species, Picornaviridae family. There are 3 serotypes of poliovirus capable of causing polio in humans: poliovirus 1, poliovirus 2, and poliovirus 3 [9]. Poliovirus is highly contagious and can be spread through the fecal-oral route or through an infected person spreading droplets by cough or sneeze. Although there is no cure for polio, the disease can be prevented through vaccination using either the live-attenuated OPV, inactivated poliovirus vaccine, or a combination of the 2 [10]. OPV is the most widely used poliovirus vaccine globally. This vaccine enables the recipient to produce antibodies as well as providing immunity in the gut, which is a key site of viral replication. Although OPV has proven to be effective in halting the transmission of polio, the vaccine's ability to mutate can result in vaccine-derived neurovirulent and transmissible strains [10]. Vaccine-derived polioviruses (VDPVs) are classified based on >1% divergence in the VP1 region for types 1 and 3 (equating to 10 mutations) and >0.6% divergence in the VP1 region for type 2 [11, 12]. VDPVs can occur in immunocompromised persons (termed iVDPV), may circulate through communities resulting in outbreaks (cVDPV), or can occur without sufficient information to classify, termed ambiguous. In rare cases, immune competent or incompetent persons receiving OPV may experience adverse events resulting in VAPP. The global polio eradication initiative defines VAPP as a “one-time case of paralytic polio that usually occurs with the first dose of OPV, with no risk of spread to others” [11, 12].

In this report, we describe a case of an infant with primary SCID complicated by VAPP. The continuing accumulation of mutations in the viral protein region (VP1) over time (from 8 to 13 in the CSF) resulted in the virus eventually being classified as an iVDPV, although the maximum number of mutations in stool was 8 [13]. These sample-specific results show compartmentalization of the virus, with continued viral evolution in the CSF, despite high-dose intravenous immune globulin, the full course of pocapavir, and no further viral shedding in the stool. Although the virus was detected in different body compartments, the time frame of mutations is consistent with the molecular clock of VP1 region.

There was insufficient poliovirus recoverable from CSF for analysis of mutations known to be associated with pocapavir resistance [14]. There were no positive results in cell cultures inoculated with CSF despite 2 attempts [15, 16]. Following stem cell transplant, onset of whole-body fasciculations suggests that symptoms may have resulted from immune reconstitution and engraftment of new lymphocytes with reaction against poliovirus in the central nervous system—an observation seen to occur previously with other diseases [17, 18]. There were no additional follow-up CSF samples.

Over the past 15 years, there have been 3 reported cases of iVDPV in South Africa, all of which were attributed to serotype 3. One patient in Johannesburg was diagnosed in 2018 with major histocompatibility complex class 2 deficiency, whereas the other 2 were diagnosed with X-linked agammaglobulinemia syndrome in Johannesburg (2011) and Cape Town (2018) [19, 20]. South Africa is 1 of few OPV-using countries with good laboratory facilities to diagnose immune deficiency, at least in urban centers [21]. Treatment options for iVDPV patients include increased doses of intravenous immunoglobulin, hematopoietic stem cell transplant, and/or experimental antiviral drugs such as pocapavir. With regard to the South African reported cases, the patients were treated with high-dose intravenous immunoglobulins and pocapavir, respectively [19, 20].

Wild poliovirus types 2 and 3 were declared eradicated in 2015 and 2019, respectively. Since the global withdrawal of Sabin poliovirus type 2 in 2016, oral polio vaccine contains only Sabin poliovirus types 1 and 3. The African region was declared free of wild poliovirus in 2020. However, countries in the region continue to use bivalent OPV on account of this vaccine's ability to induce gut immunity and prevent infection and transmission with non-type 2 cVDPV strains and the risk of importation of wild poliovirus type 1 from Pakistan or Afghanistan. Ongoing wild poliovirus type 1 outbreaks in African countries highlight the importance of maintaining herd immunity to polio among African children through continued vaccination with bivalent OPV.

Children with primary immune deficiency are susceptible to otherwise harmless infections. Adverse events following immunization with live vaccines can serve as indications for early investigation and treatment of immune deficiency. Early bone marrow transplant can be lifesaving. In a recent study conducted by Singanayagam et al, the authors describe a case of iVDPV in 2 asymptomatic male children with primary immune deficiency in the United Kingdom [22]. Although the patients successfully cleared the infection through high-dose intravenous immunoglobulin and hematopoietic stem cell transplantation, respectively, in the absence of active polio surveillance, the cases would have been missed.

Immunodeficient patients with VAPP may excrete iVDPV long-term [23]. In our case, a delayed diagnosis may have led to the accumulation of more mutations, with a potentially fatal outcome. Heightened suspicion of primary immune deficiency is required in patients presenting with acute flaccid paralysis. Thorough workup for primary immune deficiency is often unavailable in low- and middle-income settings but the diagnosis may be suspected by widely available tests including a good clinical examination, exclusion of HIV, a full blood count including quantification of total lymphocytes, and CD4 T cell count. Low total immunoglobulin G is supportive of the SCID diagnosis [24]. Early diagnosis and referral to specialized centers may lead to effective interventions.

## Notes

***Patient consent statement***. The patient's written consent was obtained for this study. The design of this work has been approved by the Human Research Ethics Committee at the University of the Witwatersrand, reference: M220966.
